# Investigation on the Electrochemical Deposition of Nanocrystalline Zinc with Cationic Polyacrylamide (CPAM)-ZnSO_4_ Electrolyte

**DOI:** 10.3390/mi12091120

**Published:** 2021-09-17

**Authors:** Xiaolei Chen, Jiasen Chen, Jiajun Zhu, Tianyu Cai, Zhongning Guo

**Affiliations:** 1 State Key Laboratory of Precision Electronic Manufacturing Technology and Equipment, Guangdong University of Technology, Guangzhou 510006, China; znguo@gdut.edu.cn; 2 Guangzhou Key Laboratory of Nontraditional Machining and Equipment, Guangdong University of Technology, Guangzhou 510006, China; chenjiasen2021@163.com (J.C.); 15779896351@163.com (J.Z.); caitianyu1104@163.com (T.C.)

**Keywords:** electrochemical deposition, nanocrystalline zinc, additive, cationic polyacrylamide (CPAM)

## Abstract

The electrochemical deposition of nanocrystalline zinc has high potential to deposit zinc coatings, which have improved wear and corrosion properties compared to conventional coating methods. Conventionally, two or more additives are used in the electrolyte for the formation nanocrystalline zinc; these electrolyte components are complex, and their maintenance is inconvenient, making it unstable and not suitable for industrial scale production. This paper proposes an electrochemical deposition technique for nanocrystalline zinc using a ZnSO_4_ solution with cationic polyacrylamide (CPAM) as the unique additive. The results reveal that the cationic degree of CPAM has a significant influence on the deposition process and that the cationic degree of 20% enhances the electrolyte conductivity and improves the density of the deposited coating. The concentration of CPAM affects the electrolyte viscosity and conductivity. CPAM with a concentration of 20 g/L could simultaneously improve the electrolyte conductivity and maintain the viscosity at a low value, which promotes the formation of a bright deposited coating with a grain size of 87 nm. Additionally, the current density affects the grain structure of the deposited coating. With a current density of 0.5 A/dm^2^, a dense coating with lamellar grains and a grain size of 54.5 nm was obtained, which has, and the surface roughness was reduced to 0.162 μm. Moreover, the corrosion resistant property of the deposited coating was also improved.

## 1. Introduction

Due to excellent corrosion resistance, zinc and zinc alloys are widely used as protective coatings in many industrial products (such as fasteners, automobile bodies, and aerospace parts) so as to improve corrosion resistance [1,2,3]. Electrochemical deposition is a popular method used to manufacture corrosion-resistant coatings.

It has been reported that the nanohardness of zinc coating prepared with a nano-scale grain size was about three times greater than the nanohardness of a zinc coating prepared with a micro-scale grain size. At the same time, both the wear rate and corrosion rate of nanocrystalline zinc coatings were significantly reduced compared to those of coarse-grained zinc coatings [4,5,6]. The above research works indicate that the electrochemical deposition of nanocrystalline zinc is a promising method to improve deposition properties.

Nowadays, acid chloride, alkaline zincate solutions, and the sulfate types of electrolytes are frequently used in the electrochemical deposition of zinc [7,8,9], and the use of different additives in the solution has been investigated to prepare nanocrystalline zinc. Ramanauskas et al. [10] investigated the pulse electrochemical deposition of nanocrystalline zinc in alkaline cyanide-free solutions with organic additives. The results indicated that the pulse current has a significant effect on the properties of the deposited coating. With pulse current, nanocrystalline zinc with a grain size of 30–60 nm was well prepared, and the corrosion behavior was significantly improved compared to that with direct current. Kenta et al. [11] investigated the effect of organic additives on the electrodeposition behavior of Zn in an alkaline zincate solution. A straight-chain polymer composed of a quaternary ammonium cation (PQ) and quaternary ammonium salt with a benzene ring (QA) was added to the solution. The results indicated that with the addition of PQ and QA, the grain size of the zinc coating was decreased, and the coating surface became smooth at the same time. Combining the advantages of organic additives and pulse electrochemical deposition, Seber et al. [12] prepared a nanocrystalline zinc coating with a grain size of 56 nm with a chlorate solution containing a mixture of polyacrylamide and thiourea. Additionally, the microhardness increased and was approximately eight times higher than that of pure polycrystalline zinc. In the work of Muralidhara et al. [13], the electrochemical deposition of nanocrystalline zinc was conducted with an acid zincate solution containing a newly synthesized condensation product (thiamine hydrochloride and furfural), which made the nanocrystalline deposition more uniform, and the grain sizes of the deposition ranged from 20–22 nm. Meng et al. [14] prepared a nanocrystalline zinc coating with a grain size of 21.5 nm using a citric acid solution, and the corrosion property was significantly enhanced. Compared to the other solutions, sulfate solutions have a deposition property that is relatively better due to the nonpolluting nature of its constituents. Nayana et al. [15] deposited a bright zinc coating using a sulfate solution with cetyltrimethylammonium bromide as an additive and with valine and veratraldehyde as a condensation product, and the maximum grain size of the deposition was 50–55 nm; the adhesion, ductility, microhardness, and corrosion resistance properties were improved at the same time.

The above studies from the literature show that the additives in the electrolyte play a major role in the grain refinement of the deposited coating, and two or more additives are usually added into the electrolyte. Furthermore, the current density, temperature, and electrolyte pH also affect the deposition process. Thus, it is difficult to analyze the influence of each parameter on the deposition of a nanocrystalline zinc coating. Moreover, the electrolyte components are complex, and the maintenance is inconvenient, which makes it unstable and not suitable for industrial production.

In the previous studies from the literature, researchers investigated the electrochemical deposition of nanocrystalline zinc coatings with unique additives, such as polyacrylamide (PAM), acrylamide (AM), formamide, acrylic acid, and polyacrylic acid [16]. The results indicated that compared to other additives, PAM could promote a higher nucleation rate and smaller grain size due to the synergistic adsorption of its polymer chain and amine group, which is beneficial for increasing the free energy to form new nuclei; however, the PAM was added in a small amount of 1 g/L in their research, and the effect of the PAM properties (the type, cationic degree, and concentration) on the deposited coating was not further investigated. This paper investigated electrochemical deposition of a nanocrystalline zinc coating using a ZnSO_4_ solution with cationic polyacrylamide (CPAM) as the unique additive. The performance of the electrolyte with added CPAM was measured, including the viscosity and conductivity. Deposition experiments with different cationic degrees and concentrations of CPAM were performed to investigate their influence on the current density, deposition surface quality, and grain size. Finally, with the optimized cationic degree and concentration of CPAM determined, nanocrystalline zinc coatings with different grain sizes and structures were deposited with different current densities, and their corrosion properties were measured.

## 2. Materials and Methods

A glass beaker was put on a temperature controlled magnetic stirrer as a reaction vessel. A direct-current (DC) power supply was employed with a zinc plate as an anode and a copper plate (substrate) as a cathode. The dimensions of the plate were 20 mm × 20 mm × 0.5 mm; both anode and cathode plates were immersed in the electrolyte, and the distance between the anode and cathode was set to 25 mm. Before electrochemical deposition, the substrate was subjected to ultrasonic cleaning with acetone (1 min) and diluted sulphuric acid (30 s) successively to remove the grease and oxidation film. It was subsequently cleaned with deionized water, and the substrate was immersed in the electrolyte for electrochemical deposition. In order to investigate the influence of CPAM on the electrochemical deposition, the electrolyte solution mainly consisted of ZnSO_4_, deionized water, and CPAM. After adding CPAM into the electrolyte solution, the solution was stirred for 30 min to make the CPAM dissolve completely. As the properties of CPAM may change at high temperatures, the temperature was set to room temperature (25 °C) in this experiment.

In the pilot test, the effect of the concentration of ZnSO_4_·7H_2_O ranging from 10 g/L to 50 g/L on the deposition process was investigated. It was observed that the deposition quality with the concentration of 40 g/L was better than that with the other concentrations, so the concentration of ZnSO_4_·7H_2_O was set to the constant of 40 g/L in this experiment. In addition, with CPAM added into the electrolyte, the range of the operating voltage was from 0.1 V to 0.5 V, and the current density ranged from 0.1 A/dm^2^ to 0.8 A/dm^2^ (0.004 A to 0.032 A). Thus, in Section 3.1 and Section 3.2, a median voltage of 0.3 V has been applied to analyze the differences in the deposition quality and the current density with different cationic degrees and concentrations of CPAM. After the cationic degree and concentration of CPAM were optimized, current densities ranging from 0.125 A/dm^2^ to 0.625 A/dm^2^ were used to investigation their effect on the grain size of the deposited coating. In the experiment, with the deposition duration increased to 15 min, CPAM will adhere to the deposited coating, hindering the continuous deposition. Thus, the deposition duration was set to 10 min in the experiment. The detailed experiment parameters are listed in Table 1.

In the experiment, a viscosity cup (QND-4, Shenzhen Anda Instruments CO., LTD., Shenzhen, China) was used to measure the viscosity of the electrolyte with different concentrations of CPAM. A conductivity meter (DDS-307, Shanghai INESA Scientific Instrument CO., LTD., Shanghai, China) was employed to measure the conductivity of the electrolyte with different concentrations and cationic degrees of CPAM. The surface morphology of the deposited coating was examined with a scanning electron microscope (S3400N, Hitachi, Chiyoda city, Japan), and the grain size was calculated using the line intercept technique on the SEM micrographs. The surface roughness of the deposited coating was evaluated using a confocal laser scanning microscope (CSLM, Olympus LEXT OLS4000, Shinjuku city, Japan). The electrochemical corrosion tests of the deposited coatings were conducted using an electrochemical workstation with a three-electrode structure (CHI-750D, Zennium, Germany) in a 3.5% NaCl solution (Guangdong Guanghua Sci-Tech CO., LTD., Shantou, China). The deposited coating surface (20 mm × 20 mm) was set as the working electrode, and a platinum plate and a saturated calomel electrode were used as the auxiliary electrode and reference electrode, respectively. All the of the tests were conducted with a scanning rate of 1 mV/s.

## 3. Results and Discussion

### 3.1. The Effect of Cationic Degree of CPAM on the Deposition Process

In general, the cationic degree of CPAM ranges from 10% to 70%, and a higher cationic degree means that CPAM has a more positive charge. In order to investigate the effect of different cationic degrees of CPAM on the deposition process, contrast experiments were performed with cationic degrees of 0 (no CPAM), 20%, 40%, and 60%. The CPAM concentration was maintained with 10 g/L, the applied voltage was set to 0.3 V, and the other parameters were set as shown in Table 1.

**Figure 1:**
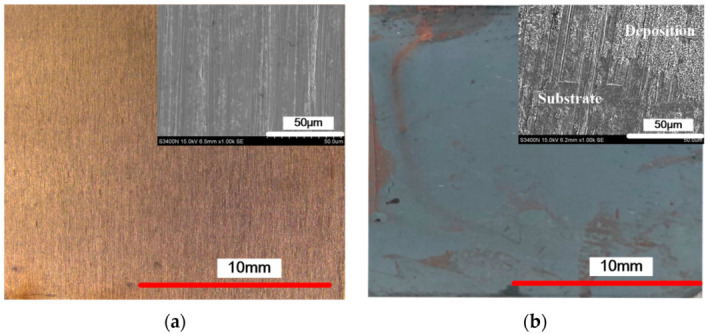

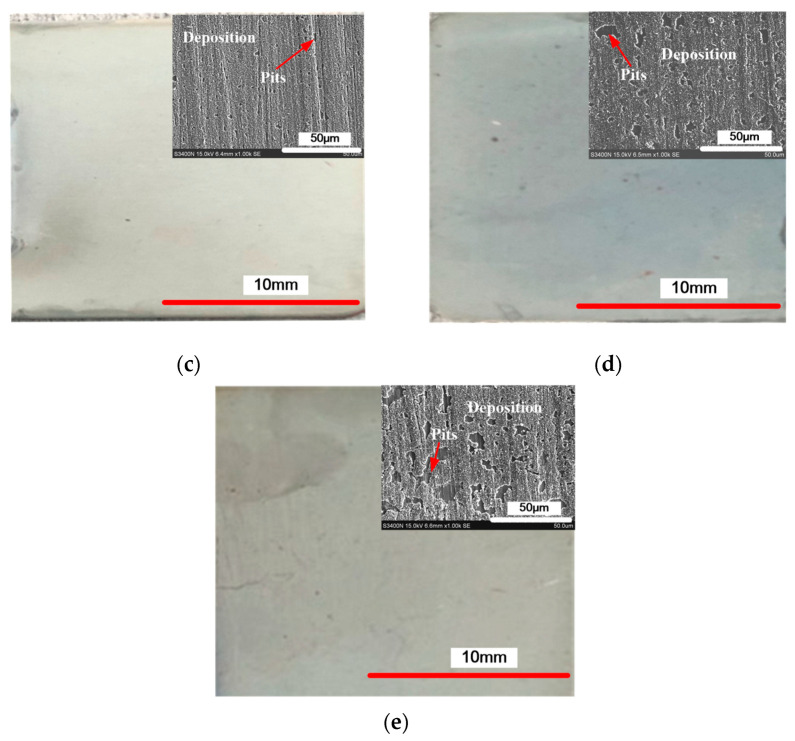
The surface morphology of the deposited coating with different cationic degrees. (**a**) Before deposition; (**b**) deposition without cationic polyacrylamide (CPAM); (**c**) cationic degree of 20%; (**d**) cationic degree of 40%; (**e**) cationic degree of 60%.

**Figure 2:**
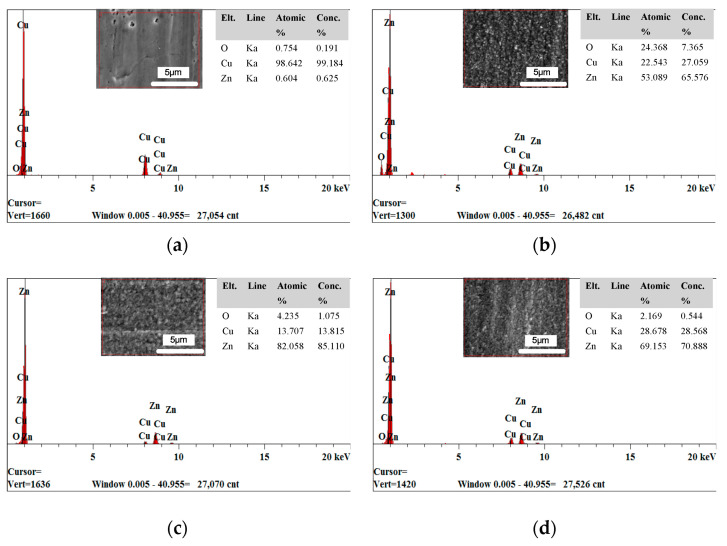

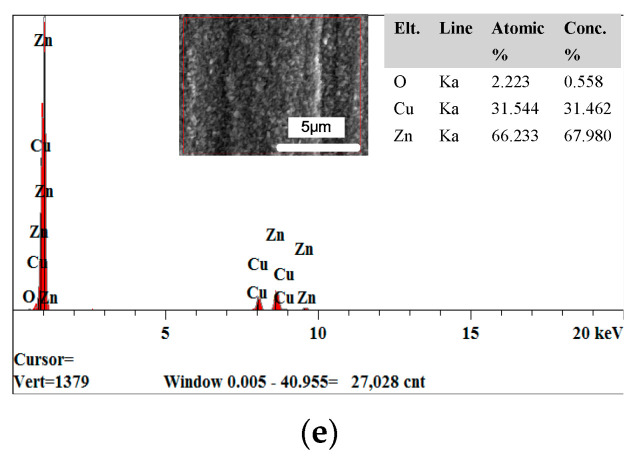
The energy dispersive spectrometer (EDS) results of the deposited coating with different cationic degrees. (**a**) before deposition; (**b**) deposition without CPAM; (**c**) cationic degree of 20%; (**d**) cationic degree of 40%; (**e**) cationic degree of 60%.

Figure 1 shows the surface morphology of the deposited coating with different cationic degrees, and Figure 2 shows the energy dispersive spectrometer (EDS) results of the deposited coating with different cationic degrees. The substrate surface before deposition is different from how it is after deposition (Figure 1a). Figure 1b shows the substrate deposited without CPAM in electrolyte; some regions of the substrate were exposed, and no zinc was deposited. The EDS results show that the Cu and Zn content was 22.543 and 53.089 at.%, respectively (Figure 2b), showing an obviously uneven deposition. When the electrolyte was added with CPAM, the whole surface of the substrate was deposited with zinc, and no specific region was exposed. Furthermore, the SEM image shows that the quality of the deposited coating changed with the different cationic degrees of CPAM. With a cationic degree of 20%, the deposited coating densified, and only a small number of pits exist on the deposited surface (Figure 1c), and the Zn content increased to 82.058 at.% (Figure 2c). With the cationic degree increased to 40%, the size of pit became enlarged, the distribution became wider at the same time (Figure 1d), and the Zn content reduced to 69.153 at.% (Figure 2d). When the cationic degree reached 60%, the pits became more obvious, the surface quality was further deteriorated (Figure 1e), and the Zn content further decreased to 66.233 at.% (Figure 2e). These results indicate that compared to traditional electrolyte, a CPAM–electrolyte significantly improves the quality of the deposited coating. Meanwhile, the cationic degree of CPAM has an important influence on the density of the deposited coating. Compared to other cationic degrees of CPAM, the cationic degree of 20% proved useful in obtaining a relatively dense coating.

Figure 2 shows the EDS results of a deposited coating with different cationic degrees. (a) before deposition; (b) deposition without CPAM; (c) cationic degree of 20%; (d) cationic degree of 40%; (e) cationic degree of 60%.

**Figure 3:**
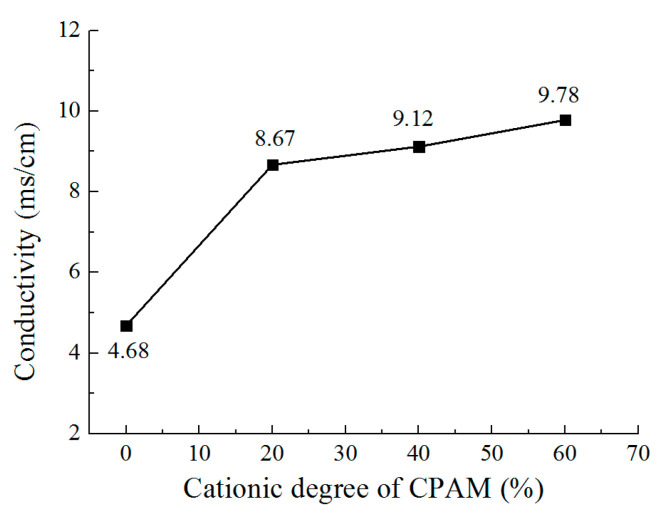
Conductivity of CPAM–electrolyte with different cationic degrees.

In order to analyze the reason for this phenomenon, the conductivity of the CPAM–electrolyte was measured, as shown in Figure 3. It can be seen that without CPAM, the conductivity of the electrolyte was 4.68 mS/cm. With CPAM added in the electrolyte, the conductivity sharply increased to 8.67 mS/cm with a cationic degree of 20%. Additionally, with the cationic degree further increased, the conductivity increased to 9.78 mS/cm at a cationic degree of 60%. This can be explained on the basis of the fact that as the number of positive charges in the macromolecule CPAM increases with the increasing cationic degree of CPAM, the conductivity of the electrolyte is also increases. Additionally, the conductivity of the CPAM–electrolyte is obviously higher than that of the traditional electrolyte. With the other parameters fixed, the current density increases with the increasing conductivity, which further enhances the cathode polarization and promotes the deposition process; thus, the quality of deposited coating is significantly improved with the CPAM–electrolyte.

**Figure 4:**
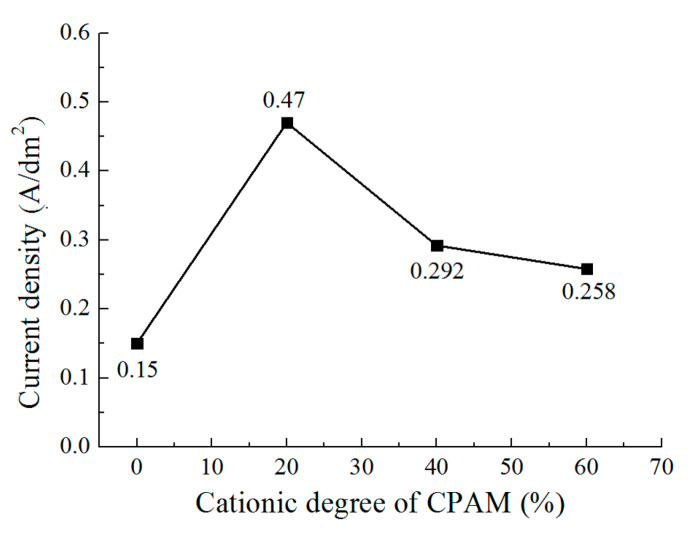
The current density during deposition with different cationic degrees.

Figure 4 shows the current density with different cationic degree of CPAM during the deposition process. It can be seen that the current density is 0.15 A/dm^2^ without CPAM. With the cationic degree of 20%, the current density increases to 0.47 A/dm^2^, while when the cationic degree further increases from 40% to 60%, the current density decreases from 0.3 A/dm^2^ to 0.26 A/dm^2^. A highest current density is observed at the cationic degree of 20%. The trend is not the same as that of the conductivity of the CPAM–electrolyte. The reason for this may be that during the deposition process, the zinc ions in the CPAM–electrolyte are transported to the cathodic substrate and then receive electrons to become zinc atoms. At the same time, the macromolecule CPAM with positive charges is also transported towards the cathodic substrate. With an increasing cationic degree, the positive charge of the macromolecule CPAM increases, more macromolecule CPAM is transported to cathodic substrate, and it is then adhered on some regions of the substrate, which forms an insulated layer and prevents further deposition on some regions. Thus, the current density is reduced with the increasing cationic degree, and the pits (defects) on the deposited coating are also enlarged at the same time. According to the experiments and analysis, it can be inferred that the CPAM–electrolyte with a cationic degree of 20% is appropriate for the deposition, which improves the conductivity of the electrolyte and the quality of the deposited coating.

### 3.2. The Effect of the Concentration of CPAM on the Deposition Quality

In order to further investigate the effect of the CPAM–electrolyte on the deposition quality, experiments with different concentrations of CPAM in the electrolyte were performed. The contrast experiments were designed with CPAM concentrations of 5 g/L, 10 g/L, 15 g/L, 20 g/L, and 25 g/L. As it has been proven that the cationic degree of 20% was appropriate for the deposition, the cationic degree of CPAM was set with 20%, the applied voltage was set as 0.3 V, and other parameters were set as shown in Table 1.

**Figure 5:**
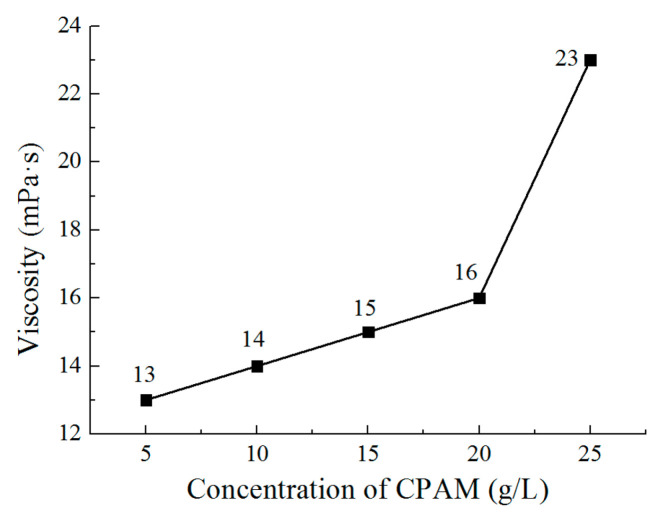
The viscosity of the electrolyte with different concentrations of CPAM.

**Figure 6:**
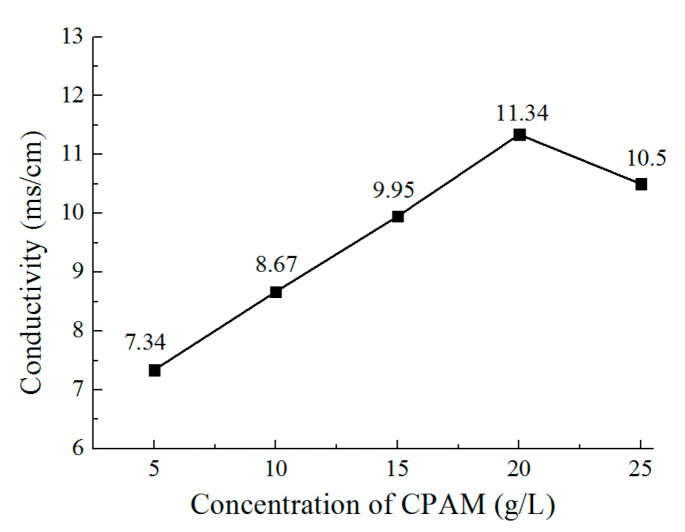
The conductivity of the electrolyte with different concentrations of CPAM.

Figure 5 and Figure 6 show the viscosity and conductivity of the CPAM–electrolyte with different concentrations, respectively. It can be observed that the viscosity increases from 13 mPa·s to 16 mPa·s when the concentration increases from 5 g/L to 20 g/L. With the concentration further increased to 25 g/L, the viscosity rapidly increases to 23 mPa·s. At the same time, the conductivity increases from 7.34 mS/cm to 11.34 mS/cm when the concentration of CPAM is increased from 5 g/L to 20 g/L. Additionally, the conductivity is then reduced to 10.5 mS/cm when the concentration is further increased to 25 g/L. This could be explained with the idea that when the concentration increases, the positive charges of CPAM increase, which improves the conductivity of the electrolyte. However, when the concentration is increased to 25 g/L, the viscosity of the CPAM–electrolyte increases rapidly, which weakens the transport of the CPAM macromolecule with positive charges in the CPAM–electrolyte; thus, the conductivity is reduced.

**Figure 7:**
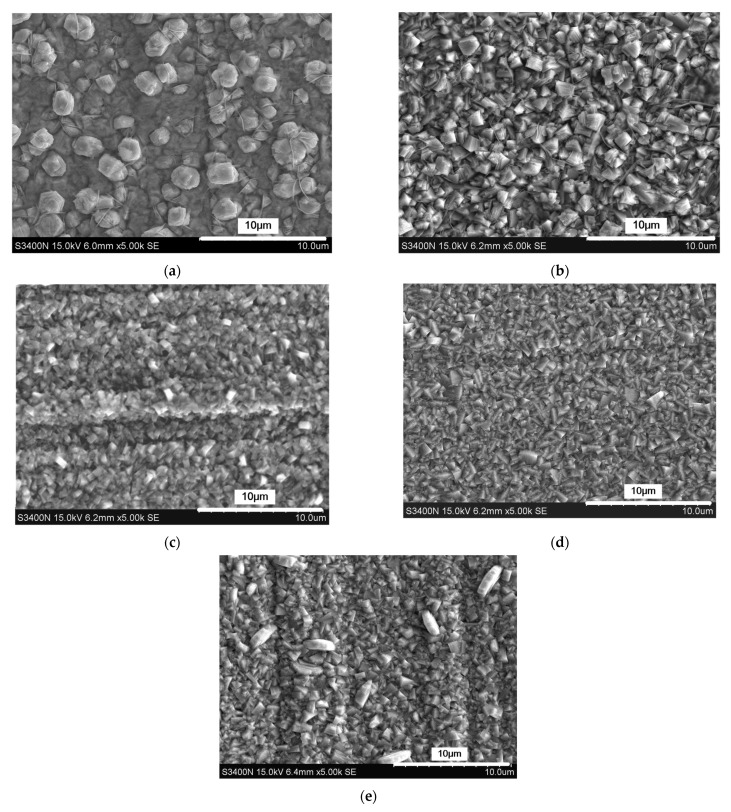
The surface morphology of the deposited coating with different concentrations of CPAM. (**a**) Concentration of CPAM = 5 g/L; (**b**) concentration of CPAM = 10 g/L; (**c**) concentration of CPAM = 15 g/L; (**d**) concentration of CPAM = 20 g/L; (**e**) concentration of CPAM = 25 g/L.

**Figure 8:**
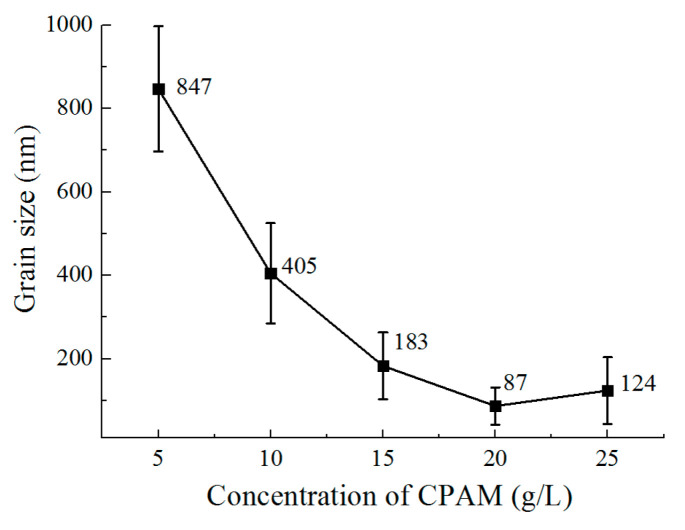
The grain size of deposition.

Accordingly, the deposition results are shown in Figure 7 and Figure 8. With a concentration of 5 g/L in CPAM, zinc deposition with spherical grains at an average size of 847 nm is formed on the surface, but the distribution of the grains is not uniform. When the concentration is increased to 10 g/L, the grains become blocky in structure with a size of 405 nm, and the distribution of the grains become uniform. When the concentration is increased from to 15 to 20 g/L, the grains are further refined, and the size reduces from 183 nm to 87 nm. However, with the concentration of 25 g/L, it can be seen that compared to that at 20 g/L, the uniformity of the grains becomes poor, and the average size increases to 124 nm.

**Figure 9:**
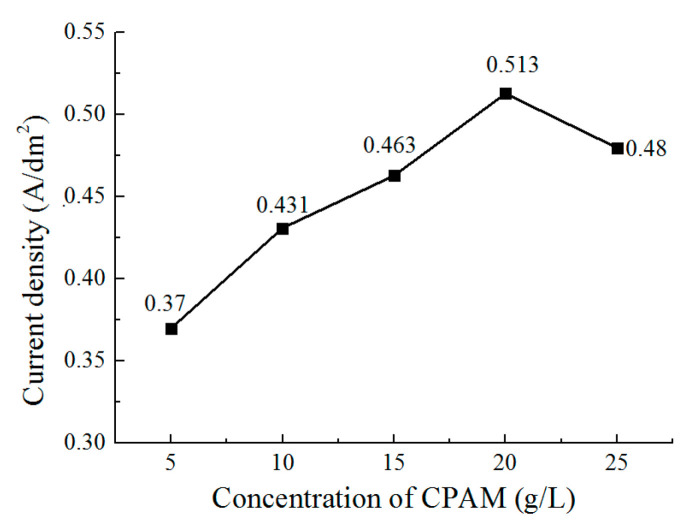
The current density during deposition.

Figure 9 shows the current density during deposition with different concentrations of CPAM. It can be observed that the current density is increased from 0.37 A/dm^2^ to 0.513 A/dm^2^ when the concentration increases from 5 g/L to 20 g/L. Additionally, then the current density reduces to 0.48 A/dm^2^ when the concentration is further increased to 25 g/L. This trend is similar to that of conductivity in Figure 6. Combined with the current density during the deposition process, the change of the grains in the process can be explained by considering that with the increase of the CPAM concentration, the conductivity and the current density of the solution is increased, which enhances the cathode polarization. High cathode polarization is useful for the generation of new nuclei, which causes the grains to become refined, which improves the surface brightness when the concentration increases from 5 g/L to 20 g/L (current density increasing from 0.37 A/dm^2^ to 0.513 A/dm^2^). In the concentration of 25 g/L, because of the increasing viscosity, the conductivity of the CPAM–electrolyte is reduced, and the cathode polarization is also weakened, which inhibits the generation of new nuclei. Accordingly, the grains become coarse, leading to a less bright surface. According to the experimental results, it can be determined that the concentration of CPAM with 20 g/L in the electrolyte is helpful in the improvement of the deposition quality.

### 3.3. The Effect of Current Density on the Deposition Quality

In Section 3.1 and Section 3.2, it has been observed that CPAM in the electrolyte increases the current density and improves the deposition quality. Meanwhile, compared to the other parameters, CPAM with a cationic degree of 20% and a concentration of 20 g/L are suitable parameters for the CPAM–electrolyte. In order to further investigate the effect of current density on the deposition quality, experiments with different current densities were performed with the optimized cationic degree and concentration.

**Figure 10:**
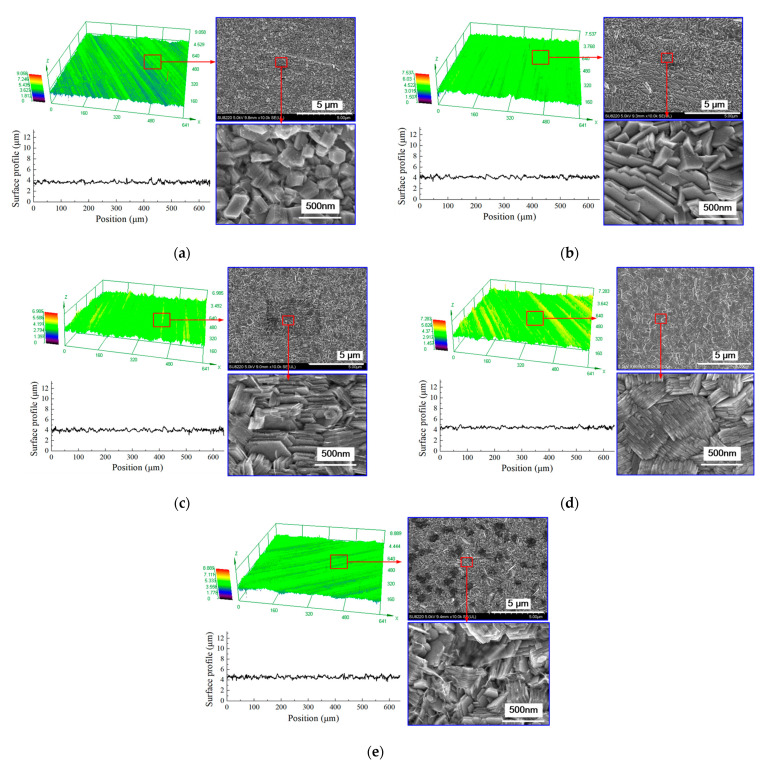
The surface morphology of the deposited coating at different current densities. (**a**) *i* = 0.125 A/dm^2^; (**b**) *i* = 0.25 A/dm^2^; (**c**) *i* = 0.375 A/dm^2^; (**d**) *i* = 0.5 A/dm^2^; (**e**) *i* = 0.625 A/dm^2^.

**Figure 11:**
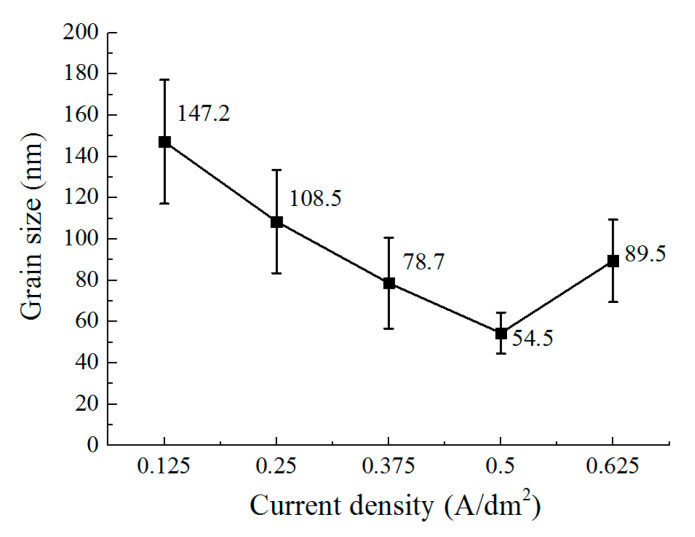
The effect of current density on the grain size.

**Figure 12:**
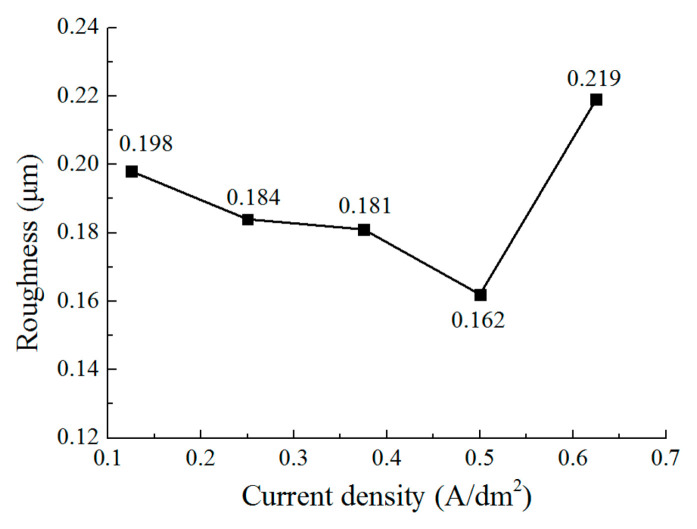
The effect of current density on the roughness.

Figure 10 shows the surface morphology of the deposited coating with different current densities. Figure 11 and Figure 12 show the grain size and roughness, respectively. It can be seen that with a current density of 0.125 A/dm^2^, a deposited coating with a blocky grain was formed (Figure 10a), the average size of which was 147.2 nm, and the roughness of the deposited surface was 0.198 μm. When the current density is increased to 0.25 A/dm^2^, the grain structure of the deposited coating changes from blocky to lamellar; the thickness of the lamellar grain is147.2 nm, and the surface roughness is 0.184 μm (Figure 10b). By further increasing the current density, the lamellar grain is further refined, and the thickness of the lamellar grain reduces to 54.5 nm when the current density is 0.5 A/dm^2^. At the same time, it can be seen that the distribution of the grains becomes more uniform and denser (Figure 10d), and the roughness reduces to 0.162 μm. Furthermore, when the current density is increased to 0.625 A/dm^2^, the size of the grain is also enlarged, and the distribution of the lamellar grains becomes tanglesome at the same time. The roughness increases to 0.219 μm. These results indicate that when at a reasonable range, the increase of current density can change the grain structure and refine the grains. However, when the current density is increased further, the deposition quality is reduced. The reason for this can be explained by the idea that the cathode polarization enhances with the increasing current density, which promotes the generation of new nuclei during deposition; thus, the grains are refined from 0.125 A/dm^2^ to 0.5 A/dm^2^. When the current density further increases to 0.625 A/dm^2^, the deposition rate is accelerated, but it is difficult for the zinc ions near the cathodic substrate to be supplied on time, which aggravates the concentration polarization. This phenomenon would not only lead to the generation of hydrogen, but it would also inhibit the generation of new nuclei; thus, the distribution of the grains becomes non-uniform, the size is coarse, and the roughness is increased as well.

**Figure 13:**
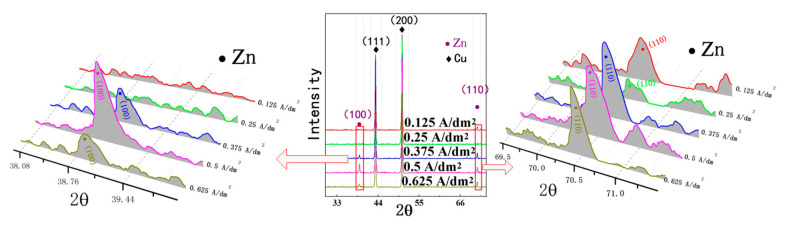
X-ray diffraction (XRD) pattern of deposited coating with different current densities.

Figure 13 shows the X-ray diffraction (XRD) patterns of the deposited coating with different current densities. Because of the thin thickness of the deposited coating, X-rays could penetrate the deposited coating easily; thus, (111) and (200) are the preferred orientations of Cu substrate in the XRD pattern. Moreover, it can be seen that (100) and (110) are the preferred orientation of the nanocrystalline zinc coating. When the current density is 0.5 A/dm^2^, it is obvious that both (100) and (110) are the preferred, which is useful to obtain a nanocrystalline zinc coating with smaller surface roughness and superior anticorrosion properties [17].

### 3.4. Tafel Polarization Curves

In Section 3.3, it was observed that the grain and roughness of the deposited coating changed at different current densities. In order to further analyze their corrosion characteristics, the Tafel polarization curves of these deposited coatings were measured in 3.5 wt% NaCl solution.

**Figure 14:**
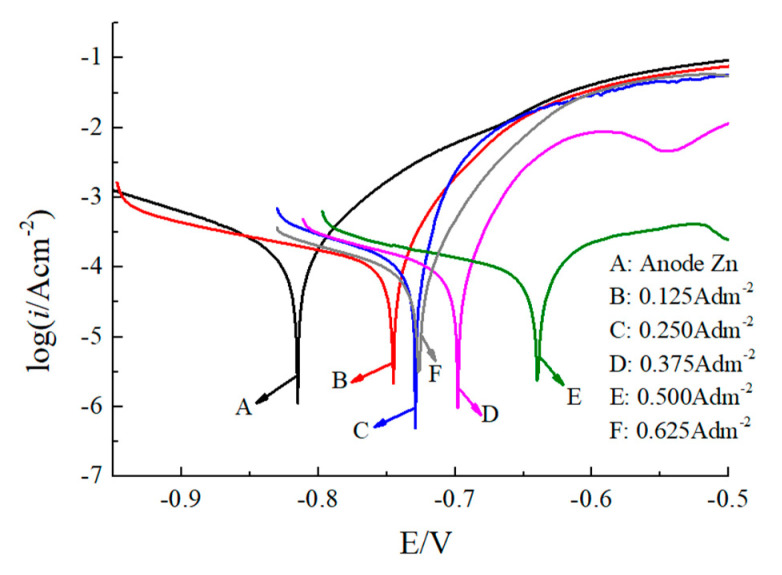
Tafel polarization curves of deposited Zn coating with different current densities.

Tafel polarization curves for the Zn coatings deposited at different current densities are shown in Figure 14, and the curve parameters, including corrosion potential (*E*_corr_) and corrosion current density (*i*_corr_), are provided in Table 2. According to Equation (1), the corrosion rate (*v*) of each sample can be calculated as follows:

(1) $$v=\frac{M}{nF}i_{corr}$$

where *M* is the atomic weight of Zn, *n* is the atomicity of Zn, and *F* is the Faraday constant.

It can be observed that the *E*_corr_ of the anodic substrate (Zn) used for deposition is −0.82 V. When the Zn coating is deposited on the cathodic substrate, the *E*_corr_ values of the Zn coating shift in a positive direction from −0.74 V to −0.64 V when the current density is increased from 0 A/dm^2^ to 0.5 A/dm^2^. Additionally, the *E*_corr_ value of the Zn coating is then reduced to −0.72 V when the current density is further increased to 0.625 A/dm^2^. A deposited Zn coating with the current density of 0.5 A/dm^2^ has the highest potential value compared to the Zn coatings deposited with other current densities, indicating that the Zn coating at this current density is more inert than those at other current densities. In addition, in Figure 14 and Table 2, it can be noted that *i*_corr_ reduces from 39.46 μA/cm^2^ to 21.18 μA/cm^2^ as the deposition current density increases from 0 A/dm^2^ to 0.5 A/dm^2^. Accordingly, the corrosion rate reduces from 0.48 g/(m^2^·h) to 0.26 g/(m^2^·h), while with the current density further increases to 0.625 A/dm^2^, and the *i*_corr_ and corrosion rate is increased to 28.25 μA/cm^2^ and 0.34 g/(m^2^·h). This indicates that with the current density of 0.5 A/dm^2^, the deposited Zn coating has higher corrosion resistant properties, and the anti-corrosion performance is superior to the Zn coatings deposited at other current densities, leading to the lowest corrosion rate. Combined with Figure 10 and Figure 11, this could be attributed to the fact that with the current density of 0.5 A/dm^2^, the distribution of the deposited grains is more uniform and denser than it is at other current densities, and the grain size is refined as well, which enhances the corrosion resistant property of Zn coating. In previous literature [13], the nanocrystalline zinc coating was deposited with an acid zincate bath, and the corrosion performance was tested in 3.5 wt.% NaCl solution. It was reported that the corrosion potential (*E*_corr_) reached −1 V, which is more negative than the corrosion potential observed in this paper (−0.64 V), indicating that the corrosion resistant property is significantly improved with the CPAM–ZnSO4 electrolyte.

## 4. Conclusions

This paper investigated the electrochemical deposition of nanocrystalline zinc with a CPAM-ZnSO_4_ electrolyte. Based on this research, the following conclusions can be obtained:

1. Compared to the electrolyte without CPAM, the CPAM–ZnSO4 electrolyte enables the electrochemical deposition of zinc at the low voltage of 0.3 V. The cationic degree of CPAM has a significant influence on the deposition process, and compared to other cationic degrees of CPAM, the cationic degree of 20% enhances the electrolyte conductivity as well as the current density, which improves the deposition density.
2. The concentration of CPAM affects the electrolyte viscosity and conductivity. Compared to other concentrations, the concentration of 20 g/L was observed to improve the electrolyte conductivity and to maintain the viscosity at a low value at the same time. A bright deposited coating with a grain size of 87 nm could be obtained at this concentration, which is better than the grain size observed with other concentrations.
3. The current density affects the grain structure of the deposited coating and the surface roughness. When the current density is increased, the grain structure changes from a blocky grain shape to a lamellar grain shape, and the grain size is also refined at the same time. With the current density of 0.5 A/dm^2^, a dense coating was deposited, the size of the lamellar grain was 54.5 nm, and the surface roughness was reduced to 0.162 μm.
4. The Tafel polarization curves show that with a current density of 0.5 A/dm^2^, the Ecorr increased to −0.64 V, and the icorr reduced to 21.18 μA/cm^2^. The corrosion resistant property of the deposited coating also improved.

## Figures and Tables

**Table 1:** Experiment parameters.

Parameter	Value
Applied voltage	0.3 V
Current density	0.125, 0.25, 0.375, 0.5, 0.625 A/dm^2^
Cationic degree of CPAM	0, 20%, 40%, 60%
Concentration of CPAM	5, 10, 15, 20, 25 g/L
Concentration of ZnSO_4_·7H_2_O	40 g/L
Deposition time,	10 min
Temperature	25 °C
Stirring rate	200 r/min

**Table 2:** Electrochemical polarization parameters of the coating with different current densities.

No.	Current Density (A/dm^2^)	*E*_corr_ (V)	*i*_corr_ (μA/cm^2^)	*v* (g/(m^2^·h))
A	Anode Zn	−0.82	39.46	0.48
B	0.125	−0.74	34.46	0.42
C	0.25	−0.725	29.22	0.36
D	0.375	−0.69	24.34	0.30
E	0.5	−0.64	21.18	0.26
F	0.625	−0.72	28.25	0.34

## Data Availability

Not applicable.
